# Annotations

**Published:** 1907-11-23

**Authors:** 


### ANNOTATIONS.

### The Early Days of Listerism.

IN his recent presidential address to the Glasgow Medico-Chirurgical Society Dr. Walker Downie gave a general review of the leading events in the life of the Society, and among these he quoted a paper read by Mr. (now Lord) Lister on April 17, 1868. This appears to have been the first public occasion on which the great surgeon gave an account of the researches on the treatment of wounds which he had been conducting in the wards of the Glasgow Royal Infirmary. In the paper in question Lister gave a full exposition of the germ theory of putrefaction, and illustrated this by the exhibition of Pasteur's experiments with flasks containing solutions of organic matter. He also described the use of carbolic acid for the destruction of germs presumed to exist in the air, and acting, as he suggested, as the exciting cause of putrefaction in wounds. In reference to the method of using carbolic acid Lister stated that after experimenting with various substances he had found a mixture of emplastrum plumbi and bees' wax, with a varying proportion of carbolic acid according to the nature of the wound, to be the most suitable external application. A number of cases were described as successfully treated by this method, and the effects of a ligature applied on the antiseptic system to the carotid artery of a horse were demonstrated. A full record of this highly interesting meeting exists in the Society's Minute Book. Lister was at the time Professor of Surgery in the University of Glasgow and one of the surgeons to the Glasgow Royal Infirmary. The discussions which followed his paper show that his teaching excited some active opposition among certain of his colleagues, though one of the surgeons, Dr. Alex. Patterson, early recognised the antiseptic method "as second only to chloroform as a boon to practical surgery." In view of the present position it is not without significance to recall the fact that Lister's system was, even years after the above date, dismissed in certain large medical schools as "a Scotch fad." And, but the other day, the President of the Medical Society, recalling Lister's advent in London, said "he remembered but would not describe it."

### Society for the Study of Disease in Children.

THIS Society met at the Medical Society's rooms on Friday the 15th inst., Dr. Geo. Carpenter in the chair. Dr. G. H. Loch read notes of a case of rheumatic hyperpyrexia in a child of six years. There had been arthritis, endocarditis, and chorea, but the evidences of these were declining rather than increasing when the temperature suddenly rose to 110° F., and this was followed by a fatal result. Emphasis was laid on the exceptional character of this development at so early an age. In the discussion, some of the speakers doubted the wisdom of extreme methods of applying cold to patients of tender years, and Dr. Frederick Taylor quoted a case in which a satisfactory result had followed free rubbing of the surface with pieces of ice. Dr. Eldon Pratt reported a case of compound fracture of the lower jaw in a child, and also an example of diffuse vaccinia following extensive ulceration occurring at the site of primary inoculation. To some of the members, however, sepsis seemed to be the more probable explanation. Among the clinical cases exhibited were two infants with congenital œdema, shown by Dr. Poynton and Dr. Sutherland. The case of a boy with ophthalmoplegia and double optic neuritis, exhibited by Dr. Carpenter, led to a discussion as to the advisability of trephining the skull, and a general opinion adverse to this proposal was expressed. In this case Calmette's reaction had been tested, at first with negative but afterwards with positive results, and one of the members quoted an identical experience on this point. Dr. Clutterbuck showed a child with intention tremor, exaggerated tendon jerks, and some spasticity of the lower limbs; and other cases were demonstrated by Mr. Russell Howard and Mr. R. Warren. The large attendance and animated discussions were in accord with former experiences, and, together with the election of a number of new members, show the value of the work which is accomplished by this Society.

### Cancer Infection and Cancer Recurrence.

MR. CHARLES RYALL, in a paper contributed to the *Lancet*, discusses the question of cancer recurrence from a very important and practical standpoint. He draws attention to the frequency with which recurrence takes place within the field of operation, as, for example, in the cicatrix and in the site of the stitches, etc.; and he expresses a doubt whether all these experiences can be satisfactorily accounted for by the plea of inadequate operation. Again, he quotes cases where after an attempted removal of cancerous tissues the disease reappears promptly and with extraordinary virulence, so that the local condition is actually worse for the surgeon's interference. For the explanation of both these sets of facts Mr. Ryall looks to the accidental infection of the wound by cancer cells at the time of operation. Such cells are necessarily liberated by the surgeon's manipulations and, being implanted in the wound, readily give rise to new growths. The development of metastases is an illustration of the infective quality of the cancer cell, and if such a cell is competent to give origin to cancerous growths when implanted in remote parts of the body, it is surely able to produce a similar result when introduced on the surface of a surgical wound. Mr. Ryall finds further support for his argument in the manifestations which often accompany cancerous growths in the abdominal cavity. There it is common to find wide dissemination of the disease, such as can only be explained by the infective character of the cancer cell and by its diffusion over the peritoneal surface. A corresponding state of matters may be produced when a chronic cancerous growth is incised, but not completely removed; cancer cells are thus able to escape and to infect the surrounding tissues. From all this there follows the necessary conclusion that the surgeon who would avoid local recurrences must take care not only to excise the whole of the cancerous tissue, but also to avoid infection of the wound which he inflicts.

